# Integrated high-precision monitoring method for surface subsidence in mining areas using D-InSAR, SBAS, and UAV technologies

**DOI:** 10.1038/s41598-024-63400-5

**Published:** 2024-05-30

**Authors:** Mingfei Zhu, Xuexiang Yu, Hao Tan, Jiajia Yuan

**Affiliations:** 1 School of Earth and Environment, Anhui University of Science and Technology, Huainan, 232001 China; 2 Coal Industry Engineering Research Center of Mining Area Environmental and Disaster Cooperative Monitoring, Anhui University of Science and Technology, Huainan, 232001 China; 3 School of Geomatics, Anhui University of Science and Technology, Huainan, 232001 China

**Keywords:** Environmental sciences, Natural hazards

## Abstract

The use of unmanned operations to monitor mining induced land subsidence is increasing. This study conducts a detailed comparative analysis of accuracy of measured ground deformation provided by Differential Interferometric Synthetic Aperture Radar (D-InSAR), Small Baseline Subset (SBAS), and Unmanned Aerial Vehicle (UAV) tilt photogrammetry with respect to levelling measurements. Based on such analysis we propose an integrated approach that combines multiple remote sensing methods to achieve a better global accuracy in the land subsidence monitoring in mining areas. Conducted at the Banji Coal Mine, this study collected subsidence data from April 10, 2021, to June 28, 2022, through D-InSAR, SBAS, and UAV techniques. After segmenting the subsidence basin into distinct zones, we qualitatively assessed each area with UAV-derived 3D models and quantitatively evaluated the precision of all applied techniques, benchmarking against leveling data. Our findings indicate that integrating D-InSAR, SBAS, and UAV technologies significantly enhances monitoring accuracy over any single method, demonstrating their combined effectiveness in different subsidence areas. Consequently, the synergistic integration of D-InSAR, SBAS, and UAV technologies, capitalizing on their complementary strengths, enables the achievement of intuitive, comprehensive, and high-precision monitoring of subsidence basins in mining areas.

## Introduction

As global industrialization escalates, the phenomenon of land subsidence resulting from the extraction of subterranean resources presents a formidable global challenge. This occurrence poses substantial risks to the safety and assets of individuals residing in mining regions and represents a significant threat to the equilibrium of global ecosystems. Notably, coal extraction, a principal energy source for humanity, is especially associated with land subsidence issues^1^. Consequently, the effective monitoring and prevention of surface subsidence in these areas are of paramount urgency.

In the context of surface subsidence monitoring within mining regions, the current standard practices predominantly involve the use of the Global Navigation Satellite System (GNSS) and traditional leveling techniques. These methodologies, while established, exhibit several drawbacks such as limited spatial resolution and confined monitoring scope. Additionally, they demand substantial time and labor resources, which constrains their efficiency and applicability in extensive, dynamic mining environments^2–4^. There is a recognized global imperative for the swift and precise tracking of surface deformations in mining areas^5^. Consequently, there is a need for a comparative analysis of the capabilities of various remote sensing technologies in monitoring surface subsidence in mining areas.

D-InSAR and SBAS, as emergent active remote sensing technologies, have gained widespread application in mining subsidence monitoring recently^6^. D-InSAR, in contrast to traditional methods, offers extensive monitoring coverage and cost-effectiveness while providing continuous spatial deformation data^7,8^. Its theoretical deformation accuracy can reach centimeter to millimeter levels. Hou et al.^9^ employed an enhanced probabilistic integral model with D-InSAR to effectively forecast and refine mining subsidence monitoring accuracy. Additionally, Wang et al.^10^ leveraged the Boltzmann function-assisted D-InSAR technique for precise surface deformation predictions in mining areas, significantly mitigating geological hazards. Their findings contribute vital insights to construction safety in these areas. Despite its merits, D-InSAR’s capacity to detect large gradient deformations is limited, and it is susceptible to various noises and atmospheric delays, posing challenges to its broader adoption in subsidence monitoring. The SBAS technique, employing multiple images and a small baseline strategy, effectively mitigates errors and noise attributable to temporal and spatial baselines, atmospheric influences, and surface scattering property variations^11–13^. This technique is adept at monitoring gradual and sustained deformation processes. Liu et al.^14^ utilized a combination of SBAS and Geographic Information System (GIS) technologies to assess the surface subsidence of a mine in Yangquan, elucidating both the intensity of subsidence in crucial areas and the associated potential damage risks. Similarly, Zhai et al.^15^ employed SBAS to track ground subsidence at Yuncheng coal mine, notably enhancing result precision and dependability by integrating corrections based on leveling data. Nonetheless, the SBAS method faces limitations in rapidly deforming regions or in areas with significant gradient changes, and its efficacy is reduced in zones characterized by dense vegetation or low coherence.

As an emerging technology in the realm of passive remote sensing, UAV inclined photogrammetry has recently gained widespread application in disaster monitoring and three-dimensional modeling. The use of UAV tilt photogrammetry presents distinct advantages compared to nadir UAV photogrammetry, particularly by offering enhanced three-dimensional visual information. This enhancement significantly improves the accuracy of 3D reconstruction for terrain and structures, particularly in complex terrains or urban environments. Notably, UAVs offer mobility, flexibility, and cost-effectiveness^16–18^. The data they produce is not only intuitive but also rich in detail, particularly when compared to InSAR technology. The enhanced precision of UAV photogrammetry, which provides pixel resolution as fine as a few centimeters, markedly exceeds the capabilities of satellite imagery, which generally offers resolutions on the order of several meters. This superiority enables the acquisition of more detailed and accurate data, crucial for applications necessitating fine-scale analysis. UAV tilt photogrammetry can achieve surface deformation monitoring accuracy to the decimeter or even centimeter level, rendering it highly suitable for monitoring significant gradient deformations in mining areas. Zhou et al.^19^ successfully employed UAVs to monitor surface subsidence at the Wangjiata coal mine, proving the technology’s efficacy in quickly gathering detailed surface subsidence basin data and accurately inverting mining subsidence prediction parameters. However, the scope of UAVs in deformation monitoring is somewhat constrained, particularly in detecting minor deformations at the edges of mining subsidence basins^19^.

The use of a single remote sensing technique for monitoring surface subsidence in mines encounters numerous challenges. Recent advances by researchers have demonstrated significant progress through the integration of multiple remote sensing methods applied to the monitoring and prediction of mining subsidence. For instance, Chen et al.^20^ conducted a thorough evaluation and calibration focusing on D-InSAR and SBAS for subsidence monitoring in mining areas. They assessed the effectiveness of these techniques across areas with varying subsidence magnitudes and introduced a methodology to enhance InSAR monitoring outcomes using leveling data. Similarly, Pawluszek–Filipiak et al.^21^ innovatively crafted a kriging-based integration approach for monitoring subsidence in mining areas, which synergistically utilizes D-InSAR and SBAS technologies. The method’s effectiveness and precision were rigorously tested and confirmed through empirical studies. Wang et al.^22^ effectively monitored surface subsidence in a plateau alpine open-pit mine by integrating UAV and Persistent Scatterets (PS) techniques. In their study of the Datong Coal Field, Xu et al.^23^ employed D-InSAR, Stacking-InSAR, and SBAS methods, finding that Stacking-InSAR exhibited high efficiency and accuracy in dense mountainous regions. Zhang et al.^24^ employed a synthesis of D-InSAR and UAV methodologies to enhance the precision of surface subsidence monitoring in mining regions. Their findings indicate that this composite technique offers greater accuracy than the use of either D-InSAR or UAV in isolation. Furthermore, Wang et al.^25^ developed a highly precise surface deformation monitoring model by amalgamating UAV, D-InSAR, and SBAS data with a probabilistic integral model, accurately capturing the dynamic surface subsidence in the mining area. Lastly, Zhu et al.^26^ applied a synergistic monitoring approach using D-InSAR and UAV, inverting the expected surface subsidence parameters via the Broyden-Fletcher-Goldfarb-Shanno (BFGS) algorithm. Their results closely aligned with the actual ground deformation patterns, validating the method’s effectiveness.

Current research on monitoring surface deformation in mining areas utilizing novel remote sensing technologies typically focuses on one or two methods to monitor or assess mining subsidence patterns and invert the expected subsidence parameters. Yet, there is often a lack of comprehensive quantitative assessment regarding the surveillance effectiveness of UAV, D-InSAR, and SBAS technologies across diverse areas of land subsidence. By considering level measurement data as the benchmark, this research compares and analyzes the capabilities of D-InSAR, SBAS, and UAV techniques in monitoring mining surface subsidence. Subsequently, this research proposes an integrated method that combines D-InSAR, SBAS, and UAV techniques. This integration aims to achieve an intuitive, comprehensive, and high-precision monitoring of the subsidence basin in mining areas.

### Study area and data

**Figure 1:**
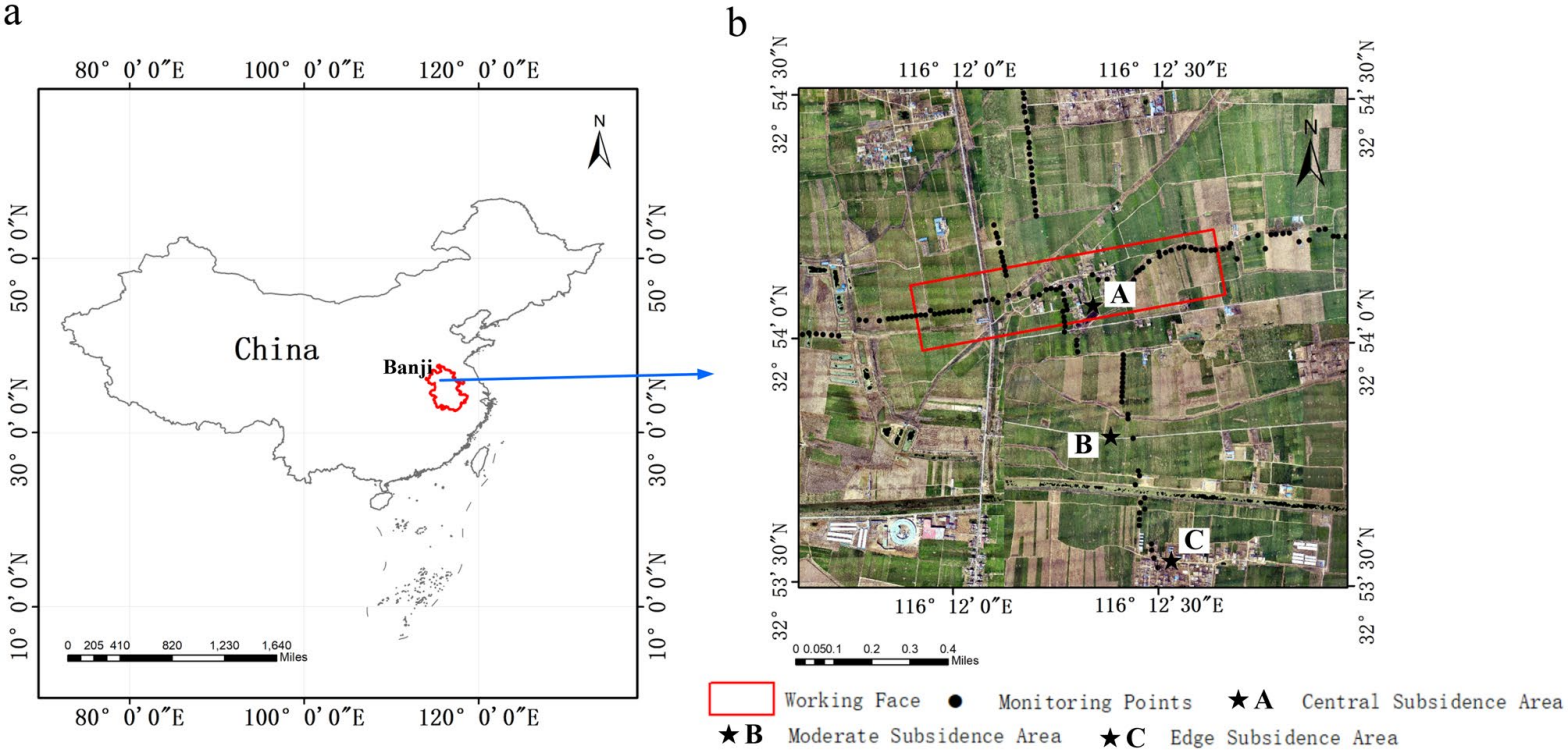
Overview of the research area: (**a**) Shows the geographic location of the research area. (**b**) The red rectangle indicates the 110,801 working face, black dots represent monitoring points, black pentagram A indicates the central subsidence area, black pentagram B indicates the moderate subsidence area, and black pentagram C indicates the edge subsidence area.

For the purposes of this research, the Banji Coal Mine, located in Bozhou City, Anhui Province, was chosen as the primary field of study. This mine is operated by China Coal Xinji Energy Company Limited and is administratively located within Huji Town, Lixin County. The 110,801 working face of this mine has a strike length of 1,237 m and an inclination width of 280 m, with the coal seam having an average depth of 767 m. For the monitoring of the 110,801 working face, 98 points were established along the strike and 92 observation points along the inclination. Figure 1 in the paper provides a visual representation of the study area’s geographic location.

In this study, the radar data employed for D-InSAR and SBAS analyses were acquired from European Space Agency’s (ESA) Sentinel-1A satellite, cover the period from the 10th of April in 2021 to the 28th of June in 2022, encompassing a total of 38 images. The pertinent parameters are detailed in Table 1. Additionally, the external Digital Elevation Model (DEM) utilized is derived from the Shuttle Radar Topography Mission (SRTM), presenting a spatial resolution characterized by a 30-m grid. In this study, UAV oblique photogrammetry was conducted using a DJI Matrice 300 RTK UAV, which was equipped with a five-lens V2 tilt camera. The data collection occurred on two separate dates: April 10, 2021, and June 28, 2022. The associated parameters are presented in Table 2. Conducted between April 10, 2021, and June 28, 2022, the leveling measurements complied with the criteria for Class III leveling precision. This protocol was meticulously followed to ensure that the elevation error in each measurement was confined to a maximum of 3.5 mm. These precise surveys formed the foundation for corroborating the accuracy of the results obtained from D-InSAR, SBAS, and UAV monitoring techniques.

**Table 1:** Parameters of Sentinel-1A data.

Satellite	Data type	Collection mode	Wavelength (cm)	Polarization	Orbit mode	Range resolution (m)	Azimuth resolution (m)	Number of images
Sentinel-1A	SLC	IW	5.6	VV	Ascending	5	20	36

**Table 2:** Parameters of UAV and camera.

UAV type	Lens type	Sidelap rate	Heading overlap rate	Ground resolution (cm)	Flight speed (m/s)	Flight altitude (m)
DJI Matrice300 RTK	PSDK 102S	70%	80%	2.5	13	160

## Methods

### Measurement of subsidence at monitoring points using D-InSAR

D-InSAR operates on the principle of analyzing phase differences within interferometric images^27^, as encapsulated by the following equation:

1 $$\begin{array}{c}\varphi ={\varphi }_{d}+{\varphi }_{f}+{\varphi }_{t}+{\varphi }_{a}+{\varphi }_{n}\end{array}$$

In this context, $\varphi$ represents the interferogram phase information acquired via D-InSAR. The deformation phase is denoted by ${\varphi }_{d}$, the flat phase by ${\varphi }_{f}$, the terrain phase by ${\varphi }_{t}$, the atmospheric phase by ${\varphi }_{a}$, and the noise phase by ${\varphi }_{n}$. Precision orbit data and external DEM data are employed to eliminate the flat and terrain phases, respectively. Similarly, a filtering method is applied to attenuate the atmospheric and noise phases, thereby enhancing the extraction of deformation phase information that is indicative of ground deformation. However, due to the periodic nature of trigonometric functions, the differential interferometric phase is confined within the range of $\left[ { - \;\pi ,\;\pi } \right]$. Therefore, it is imperative to unravel the phase to obtain the differential interferometric phase ${\varphi }_{r}$, which accurately represents the actual ground surface deformation^28,29^. The deformation along the radar line of sight can then be quantified using the following equation:

2 $$\begin{array}{c}D=\frac{\lambda }{4\pi }{\varphi }_{r}\end{array}$$

D represents the surface deformation along the radar line-of-sight, $\lambda$ is the radar wavelength, and ${\varphi }_{r}$ is the differential interferometric phase corresponding to the actual surface deformation. After obtaining the deformation along the radar line-of-sight (LOS) with D-InSAR, we convert these measurements into vertical deformation to determine the subsidence monitoring results.

**Figure 2:**
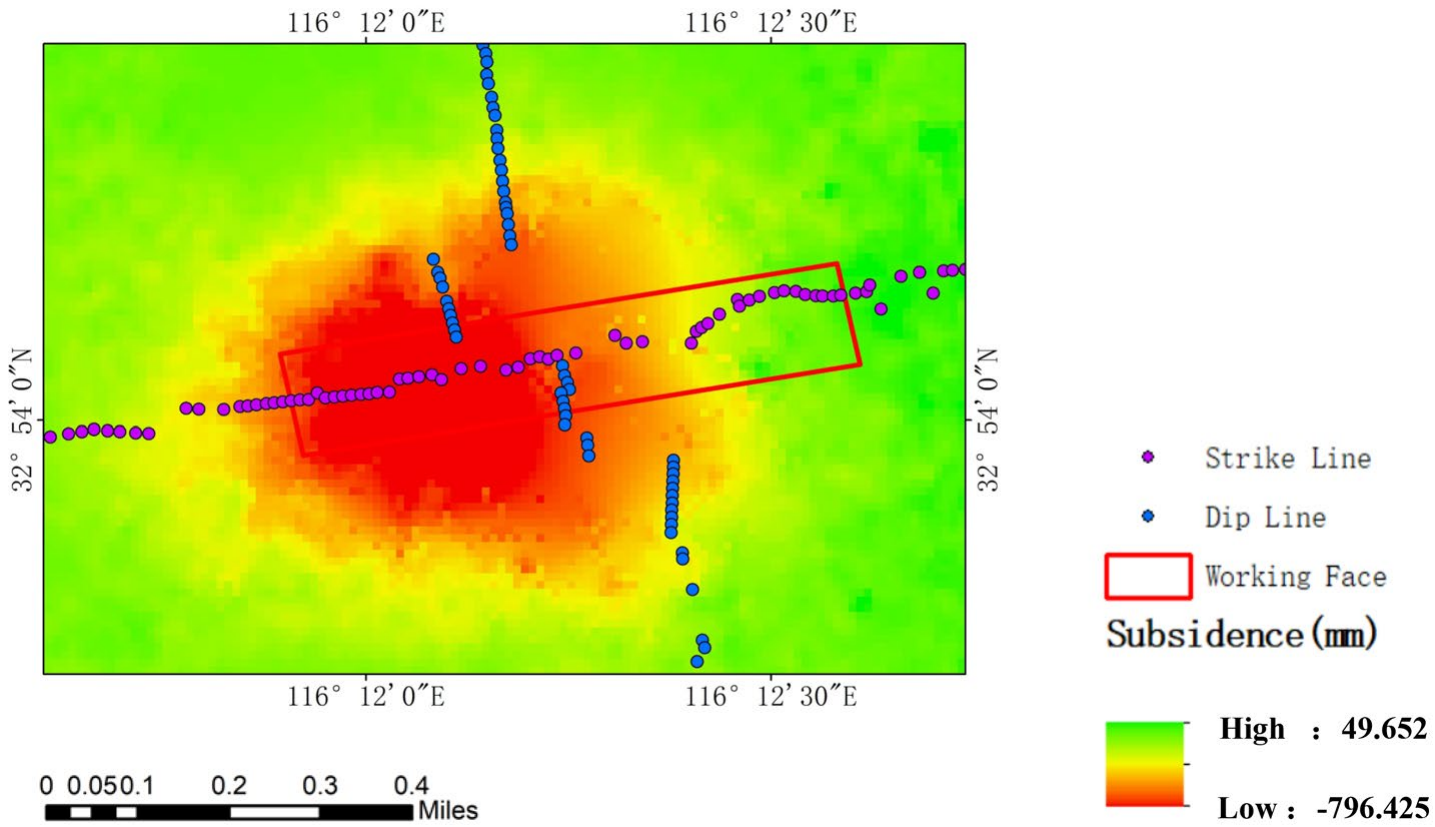
Cumulative subsidence results monitored by D-InSAR.

In this study, the data processing phase employed SARscape software, specifically version 5.6.2, which is a product developed by SARmap, an esteemed organization situated in Caslano, Switzerland. The software was operated within the framework of the ENVI platform, a widely recognized tool in the field of remote sensing analysis. To minimize the relative temporal baseline for interferometry and ensure optimal interference effects for each pair, 35 image pairs were constructed using SAR images from April 10, 2021, to June 28, 2022. The temporal baseline for all interferometric pairs was 12 days, except for two pairs covering April 10, 2021, to May 4, 2021, and May 4, 2021, to May 28, 2021, which had a baseline of 24 days. Settlement data were extracted for each time period at the monitoring points. These data were then cumulatively summed to calculate the total subsidence at each monitoring point from April 10, 2021, to June 28, 2022. Figure 2 presents the cumulative subsidence results of the mining area, as monitored by D-InSAR.

### Measurement of subsidence at monitoring points using SBAS

SBAS involves acquiring N images of the same region. These images undergo preprocessing steps such as alignment, cropping, and multiviewing to create a short baseline dataset. This dataset formation ensures high coherence in the interferometric pairs, effectively addressing the incoherence issues arising from spatial and temporal baselines. Each interferometric pair is then processed through differential interferometry, filtering, and de-entanglement to accurately derive the de-entangled phase. Atmospheric errors are mitigated using spatial and temporal filtering, enabling the accurate determination of the cumulative deformation time series relative to the first SAR image^30–32^. Ultimately, the surface Line-of-Sight (LOS) deformation detected via SBAS is transformed into vertical deformation to derive the final subsidence monitoring outcomes.

**Figure 3:**
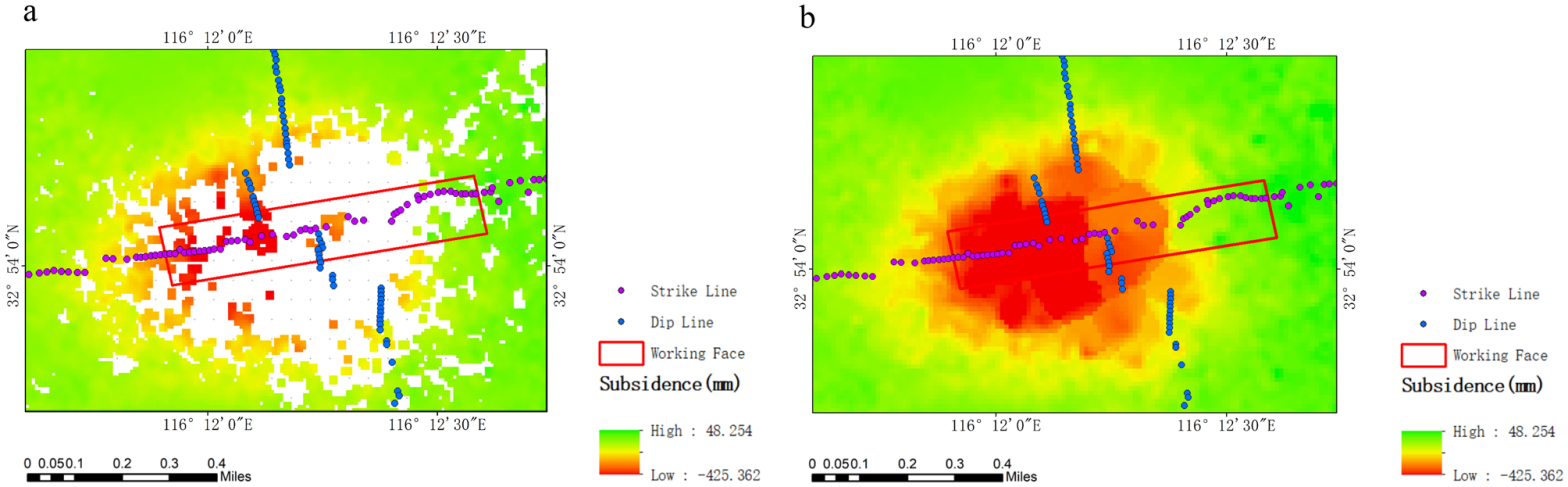
SBAS processing results: (**a**) original results; (**b**) results after kriging interpolation.

In this study, SBAS data were processed using SARscape software (version 5.6.2). The maximum temporal and spatial baselines were set at 50 days and 2%, respectively. A total of 38 SAR images from April 10, 2021, to June 28, 2022, were combined based on these thresholds to form 131 temporal differential interferometric pairs. These pairs were then processed to acquire deformation information for the study area. Due to incoherence resulting from large gradient deformation, SBAS failed to extract a sufficient number of high-coherence pixels in the central area of the subsidence basin. Consequently, it was not possible to obtain subsidence data for all monitoring points. To address this, the study employed the kriging method to interpolate SBAS results, thereby deriving subsidence data for all monitoring points, as depicted in Fig. 3.

### Measurement of subsidence at monitoring points using UAV

The essence of UAV oblique photography involves constructing high-precision route or area network models using a succession of overlapping aerial images, coupled with photogrammetric techniques and a limited number of ground image control points^33^. Central to this process is aerial triangulation, which hinges on the precise data from positioning systems and ground control points for the meticulous orientation correction of aerial images^34^. The fundamental principle of aerial triangulation rests on the geometric postulate that the photograph’s projection center, specific image points, and corresponding ground control points must align collinearly. In this methodology, each image is analyzed as an independent unit. Integration of homonymous points and ground control points across images establishes a connected regional network. Subsequently, the coordinates of densified points are precisely determined through global adjustments to this network. The core of this procedure is the covariance equation for the center projection, facilitating the construction of two corresponding error equations based on each photograph’s image point coordinates. Additionally, the six parameters required for the external orientation elements of the image are deduced via a least-squares leveling method. This series of meticulous calculations and adjustments enables the accurate determination of each densified point’s coordinates, achieving high-precision geographic mapping^35,36^.

The UAV imagery is processed to generate a point cloud dataset, encapsulating extensive 3D coordinate information of the ground surface. This data effectively delineates the ground’s shape and features, facilitating the construction of a Digital Surface Model (DSM) for the study area^37^. Subsequently, a process of real-world 3D modeling is undertaken. Here, the point cloud data are transformed into an irregular triangular mesh structure. This phase includes model optimization strategies, such as mesh simplification to enhance processing efficiency, and texture mapping. The latter involves transferring texture information from the original imagery onto the 3D model, thereby augmenting its visual realism and detail. The model undergoes rigorous validation and adjustment, culminating in the creation of a highly accurate and intricately detailed 3D model.

**Figure 4:**
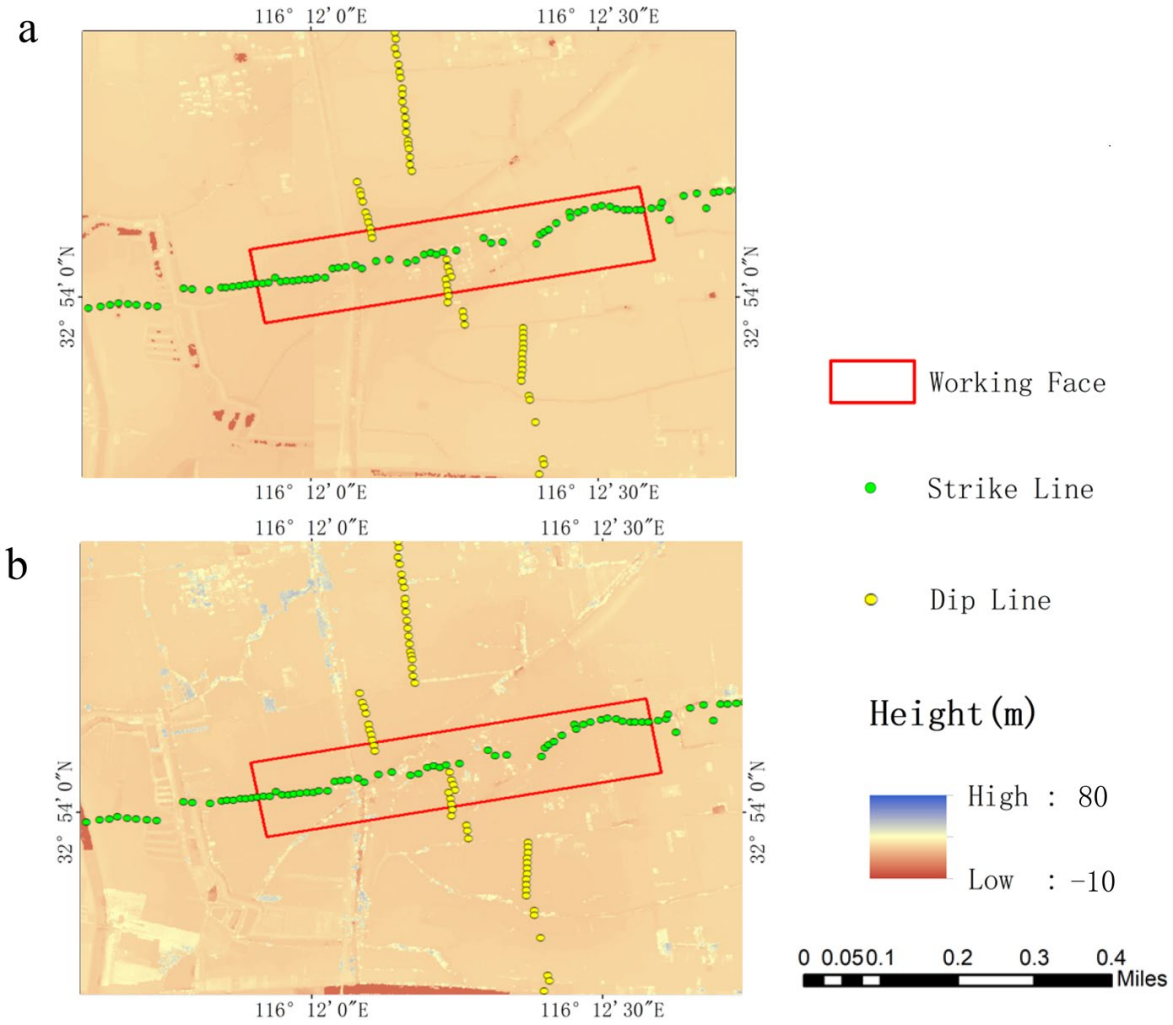
DSMs generated by UAV at two time points. (**a**) DSM generated by a UAV on April 10, 2021; (**b**) DSM generated by a UAV on June 28, 2022. After generating the DSMs, the elevation values of the monitoring points at the two time points are extracted and subtracted to determine the final subsidence amount at each monitoring point.

In this study, Bentley’s ContextCapture software (version 4.4.10) was employed for UAV data processing, resulting in the creation of DSMs and 3D models for two specific dates: April 10, 2021, and June 28, 2022. The determination of subsidence levels at specified monitoring locations during a defined temporal interval necessitated the retrieval of elevation data from the DSMs corresponding to the respective dates under consideration. These extracted elevation values were subsequently subjected to a subtraction operation, resulting in the computation of the overall subsidence magnitude occurring at these designated monitoring sites over the specified time frame, as illustrated in Fig. 4.

### Division of regions in the subsidence basin

To accurately analyze the monitoring accuracy of D-InSAR, SBAS, and UAV technologies, this study combines the principles of InSAR and UAV technologies with the patterns of surface movement and deformation in mining subsidence areas. Accordingly, the subsidence basin is divided into three zones: the central subsidence area, the moderate subsidence area, and the peripheral subsidence area. In mining subsidence research, the main influence radius is a critical concept. This study defines the outer boundary of the central subsidence area as the locations where the subsidence value reaches 0.16 ${W}_{0}$, with ${W}_{0}$ denoting the maximum subsidence. At these points, the surface exhibits maximal positive curvature. The experiment revealed that the maximum subsidence recorded at the monitoring points, determined through leveling measurements, was 2.52 m. Consequently, areas experiencing subsidence exceeding 0.4 m have been designated as the central subsidence area.

Zebker et al.^38^ observed that accurate deformation solutions become unfeasible when the deformation between neighboring interferometric image elements exceeds half the wavelength. Given that the wavelength of Sentinel 1A is 0.056 m, the maximal resolvable deformation for adjacent image elements theoretically stands at 0.028 m. By mining subsidence science standards, points with a subsidence of 0.01 m are identified as the subsidence boundary. SBAS, known for its higher theoretical accuracy than D-InSAR in detecting minor deformations at subsidence boundaries, employs multiple images and a small baseline strategy to mitigate errors and noise stemming from temporal and spatial baselines, atmospheric effects, and surface scattering characteristic variations. Consequently, in this study, regions are classified based on subsidence levels: those with subsidence below 0.028 m are identified as the edge subsidence zone; areas experiencing subsidence between 0.028 and 0.4 m are categorized as the moderate subsidence zone; and regions where subsidence exceeds 0.4 m are defined as the central subsidence zone.

**Figure 5:**
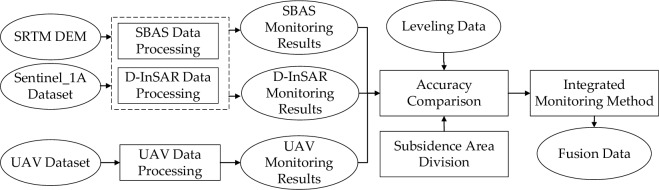
Workflow of this study.

The methodology of this study is illustrated in Fig. 5.

## Results

### Qualitative analysis of surface subsidence based on real-scene 3D models

**Figure 6:**
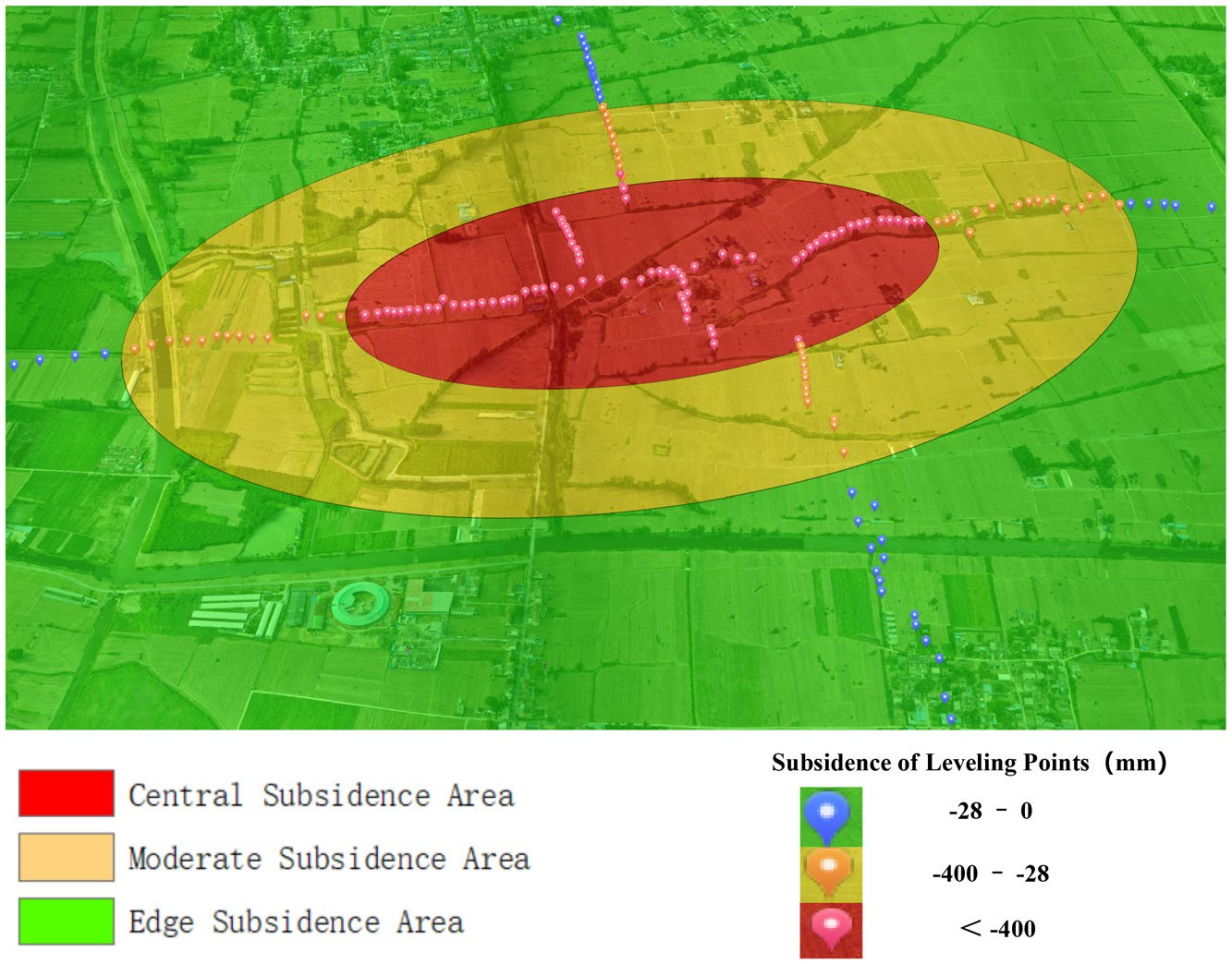
Overview of the three-dimensional model and subsidence areas division in the mining area.

The three-dimensional model of the mining zone, generated through UAV inclined photogrammetry, provides a unified spatio-temporal framework for deformation monitoring. Figure 6 presents the actual three-dimensional model of the mining area as of June 28, 2022, and illustrates the distribution of the central, moderate, and edge subsidence zones, offering a visual overview of the overall subsidence of the terrain in the mining area.

**Figure 7:**
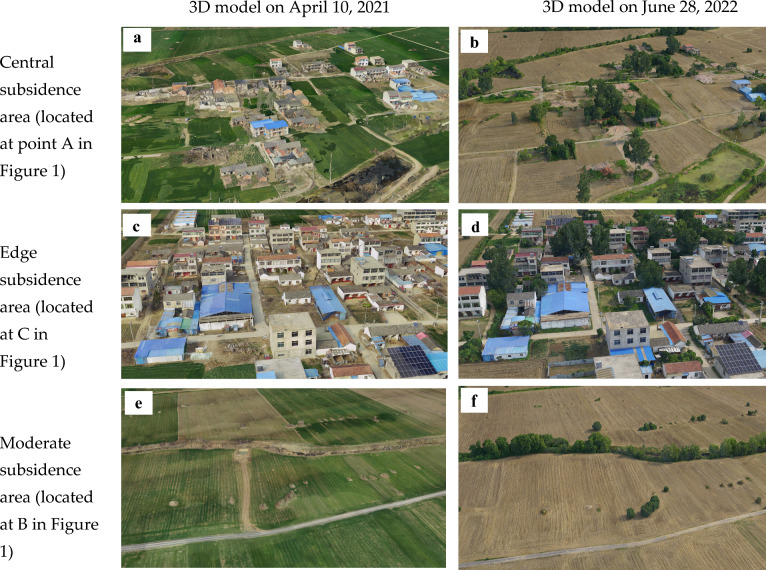
3D models: (**a**) 3D model of the central subsidence area on April 10, 2021; (**b**) 3D model of the central subsidence area on June 28, 2022; (**c**) 3D model of the edge subsidence area on April 10, 2021; (**d**) 3D model of the edge subsidence area on June 28, 2022; (**e**) 3D model of the moderate subsidence area on April 10, 2021; (**f**) 3D model of the moderate subsidence area on June 28, 2022.

Figure 7a,b illustrate the central subsidence area, where surface deformation reached approximately 2 m between April 10, 2021, and June 28, 2022. Significant changes in land cover were observed in this area. The majority of village houses suffered damage, leading to demolition. Consequently, the land cover transformed from buildings and agricultural fields to wasteland. Additionally, the extent and depth of waterlogged areas in the central part of the subsidence basin have markedly increased. Our research demonstrates the application of UAV-acquired point cloud data to overcome the challenges posed by InSAR decorrelation in areas with significant surface deformation gradients and varied surface cover. The processed UAV data provided high-resolution three-dimensional models and Digital Surface Models (DSMs), facilitating precise deformation analysis in the region of study.

The subsidence edge area, depicted in Fig. 7c,d, exhibits surface deformation of approximately 10 mm. In this periphery of the subsidence basin, due to minimal subsidence, the village structures remain largely unchanged, and the surface cover type is relatively stable. This stability allows for the extraction of high-coherence image elements, rendering the area suitable for D-InSAR and SBAS monitoring. Figure 7c,d shows a notable density of trees and houses in the area. To accurately determine the overall surface elevation of this region, it is essential to apply a filtering algorithm to the DSM that effectively removes the impact of the heights of both vegetation and buildings. In mining subsidence research, a 10 mm subsidence point is considered a boundary indicator of the surface subsidence basin. While the accuracy of UAV monitoring can achieve sub-centimeter precision, it lacks the capability to discern a 10 mm subsidence boundary. In contrast, InSAR’s sensitivity to minor deformations gives it a significant edge over UAV in monitoring the marginal subsidence areas.

Figure 7e,f illustrate the moderate subsidence area, where surface deformation measures approximately 0.4 m. Notably, the vegetation type on the surface undergoes significant changes during the subsidence period. In processing D-InSAR and SBAS data, this area’s coherence coefficient is lower compared to the edge subsidence area, resulting in relatively poorer deformation monitoring accuracy. Therefore, a quantitative analysis of the monitoring effectiveness of D-InSAR, SBAS, and UAV technologies in this moderate subsidence area is imperative, particularly in conjunction with the collected leveling data.

### Quantitative comparison of multi-source monitoring data in different subsidence areas

**Figure 8:**
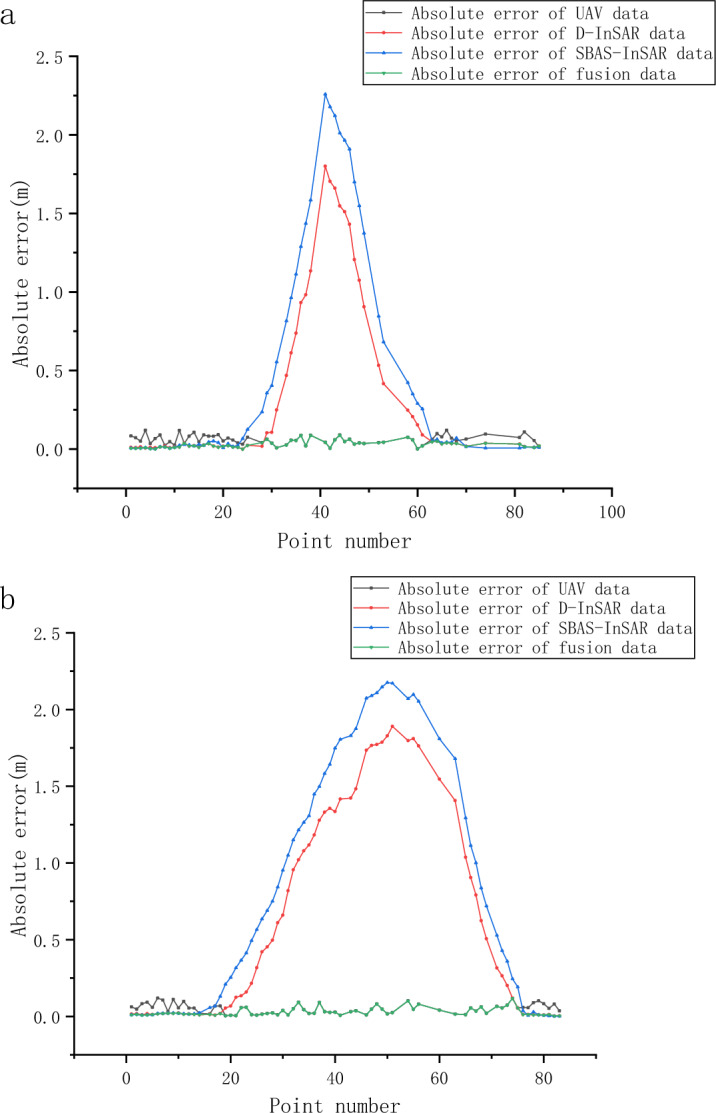
Comparison of absolute errors for multi-source monitoring data: (**a**) dip line and (**b**) strike line. The image illustrates the absolute errors in subsidence monitoring results obtained from UAV, D-InSAR, SBAS, and the integrated outcomes. The horizontal axis shows the identification numbers of the monitoring points, while the vertical axis represents the absolute errors.

This study employed SBAS, D-InSAR, and UAV technologies to obtain the subsidence measurements at monitoring points from April 10, 2021, to June 28, 2022. Focusing on three areas within the subsidence basin, this research evaluated the monitoring effectiveness of each method in different subsidence regions by comparing them against leveling data, considered as the ground truth. The evaluation was based on three metrics: maximum absolute error, mean absolute error, and root mean square error. Tables 3 and 4 present the monitoring results of the dip line and strike line in each subsidence area, respectively. Figure 8 depicts the absolute error values in the monitoring results obtained from UAV, D-InSAR, SBAS, and their integrated data, using leveling survey results as the benchmark for ground truth.

**Table 3:** Dip line monitoring results.

Area	Technique	Maximum absolute error (m)	Mean absolute error (m)	Root mean square error (m)
Central subsidence area	UAV	0.090	0.046	0.052
	D-InSAR	1.801	0.793	0.980
	SABS-InSAR	2.259	1.147	1.333
Peripheral subsidence area	UAV	0.120	0.061	0.068
	D-InSAR	0.019	0.011	0.012
	SABS-InSAR	0.015	0.007	0.008
Moderate subsidence area	UAV	0.120	0.072	0.077
	D-InSAR	0.051	0.024	0.018
	SABS-InSAR	0.185	0.050	0.070

**Table 4:** Strike line monitoring results.

Area	Technique	Maximum absolute error (m)	Mean absolute error (m)	Root mean square error (m)
Central subsidence area	UAV	0.118	0.039	0.049
	D-InSAR	1.891	0.944	1.124
	SABS-InSAR	2.176	1.198	1.368
Peripheral subsidence area	UAV	0.092	0.071	0.073
	D-InSAR	0.018	0.016	0.016
	SABS-InSAR	0.013	0.011	0.012
Moderate subsidence area	UAV	0.120	0.067	0.094
	D-InSAR	0.022	0.013	0.014

The results of the study reveal that in the central subsidence zone, the error associated with subsidence measurements obtained through UAV technology is significantly low. The Root Mean Square Error (RMSE) for UAV technology is observed to be in excess of 94% lower compared to D-InSAR, and surpasses 96% lower than that observed for SBAS. Conversely, within peripheral subsidence regions, SBAS technology demonstrates the least measurement error in subsidence detection, where its RMSE exceeds 25% lower than that of D-InSAR, and is over 83% lower compared to UAV technology. In areas experiencing moderate subsidence, the measurement error recorded for D-InSAR is the minimal, with its RMSE being substantially over 76% lower than that of UAVs and more than 63% lower than SBAS.

### Comparative analysis of the accuracy of integrated monitoring methods

**Figure 9:**
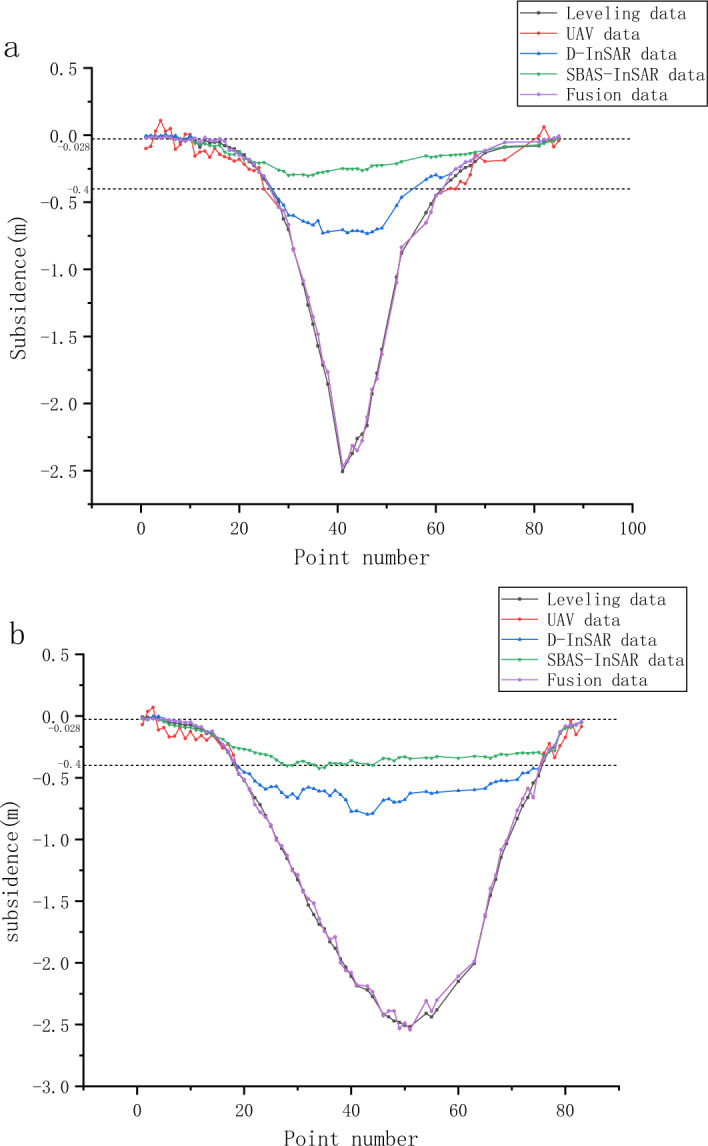
Comprehensive comparison of multi-source monitoring data: (**a**) dip line and (**b**) strike line. The image depicts the subsidence of monitoring points as measured by levelling, UAV, D-InSAR, SBAS, and the results after integration. The horizontal axis represents the identification numbers of the monitoring points, while the vertical axis indicates the amount of subsidence.

This research introduces an innovative surface subsidence monitoring methodology for mining areas, integrating D-InSAR, SBAS, and UAV technologies. Specifically, the method allocates UAV monitoring to the central subsidence zone, D-InSAR to areas of moderate subsidence, and SBAS to the peripheral zones. This comprehensive approach ensures accurate subsidence measurement across all monitored points. Figure 9 presents a comparative analysis of subsidence measurements at various monitoring points within the mining area, spanning from April 10, 2021, to June 28, 2022. These measurements were obtained using four distinct monitoring methodologies, alongside an integrated approach.

Tables 5 and 6 present the maximum absolute error, average absolute error, and root mean square error for the UAV, D-InSAR, SBAS, and fused data along the inclination and strike lines, using level monitoring results as reference values. The superiority of the fused monitoring results over individual methods is evident from these comparisons.

**Table 5:** Accuracy comparison along dip line.

Data source	Maximum absolute error (m)	Mean absolute error (m)	Root mean square error (m)
UAV	0.120	0.060	0.066
D-InSAR	1.801	0.332	0.622
SABS-InSAR	2.259	0.485	0.848
Fusion data	0.090	0.030	0.038

**Table 6:** Accuracy comparison along strike line.

Data source	Maximum absolute error (m)	Mean absolute error (m)	Root mean square error (m)
UAV	0.120	0.050	0.058
D-InSAR	1.891	0.617	0.905
SABS-InSAR	2.176	0.784	1.102
Fusion data	0.118	0.030	0.040

## Discussion

The experimental findings indicate that the monitoring techniques—UAV, D-InSAR, and SBAS—each have unique advantages and vary in effectiveness across different subsidence areas. Specifically, while UAVs are not precise in delineating the subsidence basin boundary, they provide results closest to reality with the least overall error. D-InSAR and SBAS, hindered by incoherence, struggle with large-gradient deformations and yield subsidence basins significantly different from actual observations.

In the central subsidence area, UAV monitoring exhibits the highest precision. Consequently, it can be considered that UAVs possess the most effective monitoring capability in the central subsidence area. D-InSAR successfully measured subsidence at all monitoring points within the area; however, its results exhibited significant deviations from the leveling measurements, with a root-mean-square error exceeding 1 m. Consequently, this study deduced that D-InSAR is not effective for monitoring central subsidence area. SBAS struggles to detect enough continuous high-coherence points for comprehensive monitoring. Even with Kriging interpolation applied to SBAS data, the resulting large errors suggest its unsuitability for accurate monitoring in the central subsidence area. D-InSAR outperforms both UAV and SBAS in the moderate subsidence area in terms of its lower maximum absolute error, average absolute error, and root mean square error within the monitoring results, regarding monitoring prowess. Therefore, D-InSAR is more efficacious in areas experiencing moderate subsidence. In the moderate subsidence area, the monitoring accuracy of both UAV and SBAS is relatively limited. Notably, SBAS lacks the capability to detect subsidence at all designated monitoring points within this area. In the peripheral subsidence area, the monitoring results of SBAS exhibit the least error, suggesting that SBAS possesses superior monitoring capabilities in these regions. However, the error advantage of SBAS over D-InSAR is not significantly pronounced, indicating that the actual precision enhancement of SBAS compared to D-InSAR is limited. In edge subsidence areas, the monitoring accuracy of InSAR is noticeably superior to that of UAVs, reflecting the advantage of InSAR technology in detecting minute deformations. In practical data processing, SBAS, as compared to D-InSAR, enables more efficient and convenient acquisition of long-term subsidence data and average subsidence rates.

Surface mining subsidence in the mining area is classified into four stages based on the characteristics of subsidence speed: pre-mining stability period, accelerated subsidence period, decelerated subsidence period, and post-mining stability period. For the accelerated subsidence period and decelerated subsidence period, the surface deformation gradient above the working face is large, allowing the subsidence area to be divided into: the edge subsidence zone, moderate subsidence zone, and central subsidence zone, which are monitored and integrated using the methods proposed in this study. For the pre-mining stability period and post-mining stability period, the surface subsides slowly and steadily. Due to the inability of UAV technology to detect minor deformations, it is more appropriate to use D-InSAR and SBAS technologies to monitor the initial movement of the surface and the slow, stable subsidence after mining. In terms of surface coverage, the central subsidence zone generally consists of farmland and sparse villages, which are conducive to drone monitoring; the edge subsidence zone consists mostly of industrial squares and urban areas, where the area is larger and the deformation smaller, thus obtaining more high-coherence points, making SBAS monitoring more suitable. Moderate subsidence zones, often comprising abandoned villages and farmlands, may not yield effective subsidence data from SBAS, hence overlaying the subsidence results from multiple D-InSAR monitoring sessions is a superior method.

Both InSAR and UAV technologies can obtain high-resolution and high-accuracy areal subsidence data at low cost. Apart from the difference in accuracy, the cost of InSAR technology is lower compared to UAV, making it more suitable for monitoring large-scale ground subsidence; UAV technology, compared to InSAR, is more flexible and offers a more diverse form of data, serving as a good complement to InSAR technology in the field of mining area surface subsidence monitoring. Regarding the bottleneck issue of monitoring the central subsidence area with D-InSAR and SBAS, this study has conducted ample experiments based solely on Sentinel data. In the future, attempts will be made to obtain synthetic aperture radar datasets with longer wavelengths or shorter revisit periods to further research in monitoring large-gradient deformation in mining areas.

## Conclusion

This research conducts a comprehensive evaluation of the deformation monitoring capabilities of D-InSAR, SBAS, and UAV at the Anhui Banji Coal Mine’s 110,801 working face. Through both holistic and segmented quantitative analyses, a novel approach for merging multi-source monitoring data is proposed. The major conclusions derived from this study are as follows:

Observational data reveals that subsidence and surface alterations diminish with increasing distance from the working face, consistent with established principles of ground movement and deformation associated with mining activities. Overall, the subsidence basin monitored by UAV aligns most closely with actual results, exhibiting the smallest overall error, whereas the monitoring results of SBAS have the largest overall error. In specific subsidence areas, UAV exhibits the highest accuracy in the central subsidence area, underscoring its optimal monitoring capability. In the moderate subsidence area, D-InSAR surpasses UAV and SBAS in terms of lower maximum and mean absolute errors, along with root mean square error, highlighting its efficacy. Conversely, SBAS shows minimal error in the peripheral subsidence area, indicating its proficiency there. Distinctively, D-InSAR and SBAS errors trend higher towards the center, while UAV monitoring maintains relatively stable error levels across various areas. Utilizing UAV technology for central subsidence area monitoring, D-InSAR for moderate zones, and SBAS for peripheral areas, the integrated results from these methods yield data with enhanced accuracy. The combined data’s maximum root mean square error, mean absolute error, and overall root mean square error significantly surpass the results from single monitoring methods. Hence, leveraging the synergistic strengths of D-InSAR, SBAS, and UAV technologies enables a more intuitive and precise monitoring of mining subsidence basins. This integrated approach not only facilitates unmanned monitoring but also significantly contributes to the ecological and environmental protection in mining regions.

## Data Availability

The radar data were obtained from European Space Agency’s (ESA) Sentinel-1A satellite (https://search.asf.alaska.edu/ (accessed on 15 May 2023)). The external Digital Elevation Model (DEM) utilized was derived from the Shuttle Radar Topography Mission (SRTM) (https://earthexplorer.usgs.gov/ (accessed on 15 May 2023)).
